# Evaluation of Antimicrobial Susceptibility Profile in *Salmonella* Typhi and *Salmonella* Paratyphi A: Presenting the Current Scenario in India and Strategy for Future Management

**DOI:** 10.1093/infdis/jiab144

**Published:** 2021-11-23

**Authors:** Balaji Veeraraghavan, Agila K Pragasam, Pallab Ray, Arti Kapil, Savitha Nagaraj, Sulochana Putli Bai Perumal, Karnika Saigal, Maria Thomas, Madhu Gupta, Temsunaro Rongsen-Chandola, Dasaratha Ramaiah Jinka, Jayanthi Shastri, Anna P Alexander, Roshine Mary Koshy, Anuradha De, Ashita Singh, Sheena Evelyn Ebenezer, Shanta Dutta, Ashish Bavdekar, Deepak More, Sonali Sanghavi, Raghuprakash Reddy Nayakanti, Jobin J Jacob, Anushree Amladi, Shalini Anandan, Baby S Abirami, Yamuna D Bakthavatchalam, Dhiviya P M Sethuvel, Jacob John, Gagandeep Kang

**Affiliations:** 1 Christian Medical College, Vellore, India; 2 Post Graduate Institute of Medical & Educational Research, Chandigarh, India; 3 All India Institute of Medical Sciences, Delhi, India; 4 St Johns Medical College, Bengaluru, India; 5 Kanchi Kamakoti CHILDS Trust Hospital, Chennai, India; 6 Chacha Nehru Bal Chikitsalaya, Delhi, India; 7 Christian Medical College, Ludhiana, India; 8 Centre for Health Research & Development-Society for Applied Studies, New Delhi, India; 9 Rural Development Trust Hospital, Bathalapalli, Andhra Pradesh, India; 10 Topiwala National Medical College & BYL Nair Charitable Hospital, Mumbai, India; 11 Lady Willingdon Hospital, Manali, India; 12 Makunda Christian Leprosy & General Hospital, Karimjang, India; 13 Chinchpada Christian Hospital, Nandurbar, India; 14 Duncan Hospital, Raxaul, India; 15 ICMR-National Institute of Cholera and Enteric Diseases, Kolkata, India; 16 KEM Hospital & Research Centre, Pune, India

**Keywords:** antimicrobial resistance, ciprofloxacin, India, typhoid fever, surveillance

## Abstract

**Background:**

Systematic studies to estimate the disease burden of typhoid and paratyphoid in India are limited. Therefore, a multicenter study on the Surveillance of Enteric Fever in India was carried out to estimate the incidence, clinical presentation, and antimicrobial resistance (AMR) trend. The data presented here represent the national burden of AMR in *Salmonella* Typhi and *Salmonella* Paratyphi A.

**Methods:**

Antimicrobial susceptibility testing was performed for *S.* Typhi and *S.* Paratyphi A (n = 2373) isolates collected prospectively during a 2-year period from November 2017 to January 2020.

**Results:**

Of 2373 *Salmonella* isolates, 2032 (85.6%) were identified as *S.* Typhi and 341 (14.4%) were *S.* Paratyphi A. Approximately 2% of *S.* Typhi were multidrug-resistant (MDR), whereas all 341 (100%) of *S.* Paratyphi A isolates were sensitive to the first-line antimicrobials. Among 98% of ciprofloxacin nonsusceptible isolates, resistance (minimum inhibitory concentration [MIC] >0.5 µg/mL) was higher in *S.* Typhi (37%) compared with *S.* Paratyphi A (20%). Azithromycin susceptibility was 99.9% and 100% with a mean MIC of 4.98 μg/mL for *S.* Typhi and 7.39 μg/mL for *S*. Paratyphi A respectively. Ceftriaxone was the only agent that retained 100% susceptibility. Moreover, beta-lactam/beta-lactamase inhibitors showed potent *in vitro* activity against the study isolates.

**Conclusions:**

Data obtained from this systematic surveillance study confirms the declining trend of MDR *Salmonella* isolates from India. The higher prevalence of ciprofloxacin nonsusceptibility enforces to limit its use and adhere to the judicious usage of azithromycin and ceftriaxone for enteric fever management.

## Teaser

Our study reports the antimicrobial susceptibility profile of S. Typhi and S. Paratyphi A collected across India. This represents the national data to help guide clinicians in their patient care decision-making and public health agencies in planning intervention strategies.

Enteric fever is a life-threatening systemic illness caused by *Salmonella enterica* serovar Typhi and Paratyphi (A, B, and C) [1, 2]. The infection is endemic to low- and middle-income countries, whereas the data on the incidence in these regions are limited [3]. In India, the incidence of culture-confirmed typhoid cases is approximately 377 per 100 000 populations, with an approximate case fatality rate of 1% [4]. Although most of these can be effectively treated with antibiotics, the disease management is challenged by the changing resistance profile observed in strains of typhoidal *Salmonella* [5].

Historically, the first-line agents (ampicillin, chloramphenicol, and trimethoprim-sulfamethoxazole) were the drug of choice for the management of enteric fever. However, due to the emergence of multidrug-resistant (MDR) *S.* Typhi in the 1970s, fluoroquinolones became the standard of care for the treatment of typhoid fever [6, 7]. However, since the 2000s there have been frequent reports of decreased ciprofloxacin-susceptible (DCS) *S.* Typhi in the endemic regions of South Asia and Southeast Asia [8, 9]. At this time, ceftriaxone, a third-generation cephalosporin (intravenous), and azithromycin, a macrolide (oral), are increasingly being used for complicated and uncomplicated typhoid fevers, respectively [2, 10, 11]. The combination of ceftriaxone and azithromycin has also been found to be a highly effective therapy for an initial episode of complicated typhoid fever [12].

Although ceftriaxone continues to be an effective treatment option, factors such as intravenous administration, cost, and longer duration of fever defervescence made it a less than ideal treatment alternative for typhoid management [13]. High intracellular concentrations and the subsequent release of drug from tissues and cells have made azithromycin equally efficacious [14]. However, the susceptibility of azithromycin is still debatable, because there has been a lack of guidelines for testing and interpretation until 2015. Furthermore, the clinical response to azithromycin has not been correlated with *in vitro* susceptibility to *S.* Typhi, and there is no supportive evidence to determine the interpretive criteria for testing *S.* Paratyphi A’s susceptibility to this agent [15]. The increasing use of ceftriaxone and azithromycin places selective pressure for the emergence and spread of resistant isolates [16, 17]. Recent reports of the emergence of ceftriaxone-resistant, extensively drug-resistant (XDR) *S.* Typhi and sporadic reports of azithromycin resistance further complicate the management of typhoid fever [17, 18].

Over the last few years, several surveillance studies have attempted to estimate the enteric fever burden and the antimicrobial resistance (AMR) trend from the endemic regions of India [17–21]. Although these studies have managed to capture the enteric fever incidences in tertiary care centers, the peripheral areas of the healthcare system are still untouched. To understand the contributing factors, outcomes, and available treatment options of typhoid fever in India, data on the burden of enteric fever and its antimicrobial resistance profile across India need to be generated. To fill such knowledge gaps, we undertook a multicentric study—the Surveillance of Enteric Fever in India (SEFI). As part of the study objectives, we made efforts to identify the difference in the disease and AMR burden of typhoidal *Salmonella* with respect to different surveillance settings such as at the community level (a defined catchment area) and secondary care and tertiary care hospitals in different locations within India, the results of which are presented in this study. These data would in turn aid in formulating the antibiotic policy, implementation of typhoid conjugate vaccines, prevention and control measures.

## METHODS

### Study Settings

**Figure 1.**
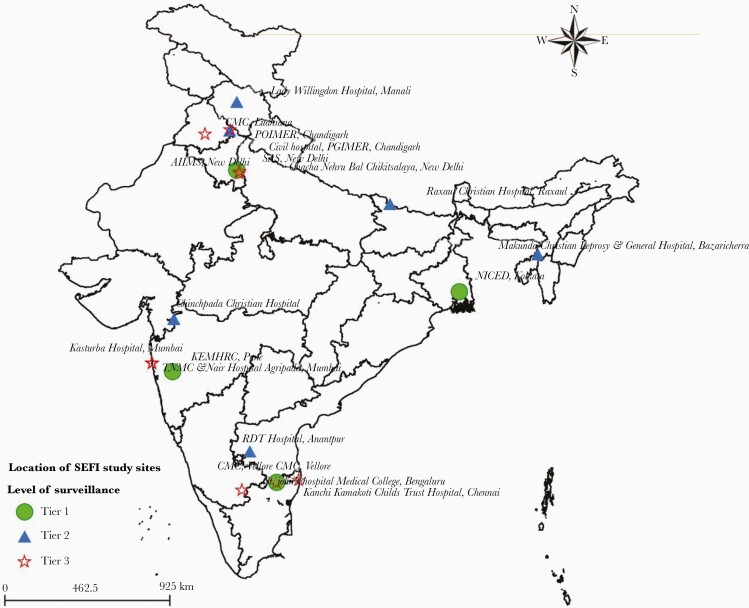
Map representing the study sites across India.

Based on the standard assessment of existing hospital facilities in India, a total of 19 centers covering community-level healthcare setting (Tier 1), secondary hospitals (Tier 2), and tertiary care hospitals (Tier 3) across the country were selected. Tier 1 included 4 sites: (1) Christian Medical College (CMC), Vellore; (2) Centre for Health Research and Development-Society for Applied Studies (CHRD-SAS), New Delhi; (3) National Institute of Cholera and Enteric Diseases (NICED), Kolkata; and (4) King Edward Memorial Hospital (KEM), Pune. Tier 2 included 6 sites: (1) Rural Development Trust (RDT), Anantapur; (2) Lady Willingdon, Manali; (3) The Duncan Hospital, Raxaul; (4) Chinchpada Christian Hospital, Chinchpada; (5) Postgraduate Institute of Medical Education & Research (PGIMER), Chandigarh; and (6) Makunda Christian Leprosy and General Hospital, Karimganj. Tier 3 included 9 sites: (1) All India Institute of Medical Sciences (AIIMS), New Delhi; (2) Christian Medical College, Vellore; (3) Chacha Nehru Bal Chikitsalaya (CNBC), New Delhi; (4) Christian Medical College, Ludhiana; (5) Kanchi Kamakoti CHILDS Trust Hospital (KKCTH), Chennai; (6) St. Johns Hospital, Bangalore; (7) Kasturba Hospital, Mumbai; (8) Topiwala National Medical College (TNMC), Mumbai; and (9) Postgraduate Institute of Medical Education and Research (PGIMER), Chandigarh. These hospitals are located in widely separated areas and represent the majority of India’s vast population (Figure 1). The laboratories participating formed the National Surveillance System for Enteric Fever in India (NSSEFI), later referred as the Surveillance of Enteric Fever in India (SEFI).

### Bacterial Isolates

During the 2-year period from November 2017 to January 2020, blood cultures positive for *S.* Typhi and *S.* Paratyphi A, isolated from the participants at each of the study sites, were collected. *S.* Typhi and *S.* Paratyphi A received at the reference laboratory (CMC, Vellore) were reidentified and confirmed by conventional biochemical tests and serotyping by slide agglutination tests according to the Kauffmann*-*White scheme [22]. Serogrouping was carried out using polyvalent O antisera (A-I). Vi, Group D, and serovar-specific STO and STH (Becton Dickinson) were used for the confirmation of *S.* Typhi and *S.* Paratyphi A.

### Antimicrobial Susceptibility

Disk diffusion (DD) testing was performed to determine susceptibility for the following agents: ampicillin (10 μg), chloramphenicol (30 μg), co-trimoxazole (1.25/23.75 μg), ciprofloxacin (5 μg), pefloxacin (5 μg), ceftriaxone (30 μg), cefixime (5 μg), and azithromycin (15 μg). In addition, the minimum inhibitory concentration (MIC) was determined for 3 agents: ciprofloxacin (0.007–4 μg/mL), ceftriaxone (0.015–2 μg/mL), and azithromycin (0.12–128 μg/mL), by reference broth microdilution (BMD) method and the test results were interpreted as per the criteria recommended by the Clinical and Laboratory Standards Institute (CLSI) guidelines [23, 24]. For a subset of randomly chosen isolates (*S.* Typhi-534; *S.* Paratyphi A-56), BMD testing was done for 3 beta-lactam/beta-lactamase inhibitor (BL/BLI) combinations piperacillin/tazobactam (0.25–128 μg/mL), cefepime/tazobactam (0.007–16 μg/mL), and cefepime/zidebactam (WCK5222: 0.007–16 μg/mL).

For setting quality control ranges for DD, American Type Culture Collection (ATCC) *Escherichia coli* 25922 was used for ampicillin (10 μg; 15–22 mm), chloramphenicol (30 μg; 21–27 mm), co-trimoxazole (1.25/23.75 μg; 23–29 mm), ciprofloxacin (5 μg; 29–37 mm), pefloxacin (5 μg; 25–33 mm), and ceftriaxone (30 μg; 29–35 mm); and ATCC *Staphylococcusaureus* 25923 was used for azithromycin (15 μg; 21–26 mm). For BMD testing, ATCC *E. coli* 25922 was used for ciprofloxacin (0.004–0.016 μg/mL) and ceftriaxone (0.03–0.12 μg/mL); and ATCC *S. aureus* 29213 (0.5–2 μg/mL) was used for azithromycin. For BL/BLIs tested by BMD testing, ie, piperacillin/tazobactam (1/4 to 4/4 μg/mL), cefepime/tazobactam (0.03/8 to 0.12/8 μg/mL), and cefepime/zidebactam (0.016–0.06 μg/mL), ATCC *E. coli* 25922 was used. Readings for clinical isolates were taken only when quality control ranges were satisfactory.

## RESULTS

A total of 2373 clinical isolates were obtained from the different participating sites after the exclusion of duplicates and nontyphoidal *Salmonella* serovars. Isolates were characterized and confirmed as *S.* Typhi (*n* = 2032) and *S.* Paratyphi A (*n* = 341). The antimicrobial susceptibility profile of total isolates to the 7 antimicrobial agents using DD tests are summarized in Table 1. Antimicrobial resistance patterns for each Tier, by the organism, shows that nonsusceptibility to fluoroquinolones is common, whereas >98% of isolates were susceptible to the other tested antimicrobials. The MDR rates were <2% for *S.* Typhi in the entire study population and predominantly confined to northern India.

**Table 1.** Antimicrobial Susceptibility Profile of *Salmonella* Typhi and *Salmonella* Paratyphi A Tested in the Present Study

Antimicrobials	%Susceptible	
	*S.* Typhi (n = 2032)	*S.* Paratyphi A (n = 341)
Ampicillin	97.3	100
Chloramphenicol	96.1	100
Trimethoprim/sulfamethoxazole	96.4	100
Ciprofloxacin	2.1	1.1
Ceftriaxone	100	100
Cefixime	100	100
Azithromycin	99.9	100

**Figure 2.**
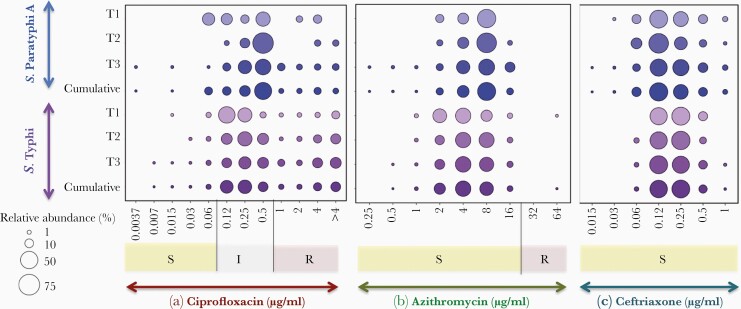
Bubble graph showing the distribution of minimum inhibitory concentration of (a) ciprofloxacin, (b) azithromycin, and (c) ceftriaxone for *S.* Typhi and *S.* Paratyphi A tested in this study.

To understand the effectiveness of current antimicrobial regimens, ciprofloxacin, azithromycin, and ceftriaxone MIC distribution for *S.* Typhi and *S.* Paratyphi A were investigated using the BMD method. Figure 2 represents Tier-wise MIC distribution for ciprofloxacin (Figure 2A), azithromycin (Figure 2B), and ceftriaxone (Figure 2C).

### Ciprofloxacin Minimum Inhibitory Concentration

For ciprofloxacin, the MIC ranged from 0.007 to >4 μg/mL with the 0.12 μg/mL–0.25 μg/mL range containing the highest proportion of *S.* Typhi isolates. It is interesting to note that the MIC range containing the highest proportion of *S.* Paratyphi A isolates was 1- to 2-fold dilution higher (0.5 μg/mL) than that of *S.* Typhi. The mean MIC was not calculated for ciprofloxacin because the endpoint MIC was not determined, and it was set up to 4 μg/mL. The cumulative MIC distribution showed 60% of the *S.* Typhi isolates were moderately susceptible (0.12–0.5 μg/mL), whereas 36% were resistant (≥1 μg/mL) to ciprofloxacin. Likewise, 79% of the *S.* Paratyphi A isolates were ciprofloxacin intermediate and 19% were ciprofloxacin resistant (Figure 2A).

Subset analysis of Tier-wise MIC variations across the community surveillance (Tier 1), secondary-care hospital surveillance (Tier 2), and tertiary-care hospital surveillances (Tier 3) was also carried out and is detailed in Table 2. Tier-wise ciprofloxacin MIC distribution for both *S.* Typhi and *S.* Paratyphi A showed a similar trend as observed in the cumulative data. It is notable that the cumulative MIC of Tier 1 showed a minor variation with a higher proportion (82.7%) of *S.* Typhi isolates being ciprofloxacin intermediate compared with Tier 2 (70.16%) and Tier 3 (54.8%). For *S*. Paratyphi A, Tier 3 had a higher proportion of resistant isolates (21%) in comparison with Tier 1 (14%) and Tier 2 (9%). In addition, there was no significant difference observed in MIC_50_ and MIC_90_ between the Tiers in *S.* Typhi except for Tier 3, where the MIC_50_ was 0.5 μg/mL, compared with 0.25 μg/mL of Tier 1 and Tier 2.

**Table 2.** Minimum Inhibitory Concentration (MIC) for Ciprofloxacin, Azithromycin, and Ceftriaxone Tested Against *Salmonella* Typhi and *Salmonella* Paratyphi A

Settings/region		Ciprofloxacin					Azithromycin					Ceftriaxone			
		%S/I/R	MIC Range (μg/mL)	MIC_50_ (μg/mL)	MIC_90_ (μg/mL)	%S/I/R	MIC Range (μg/mL)	Mean MIC (μg/mL)	MIC_50_ (μg/mL)	MIC_90_ (μg/mL)	%S/I/R	MIC Range (μg/mL)	Mean MIC (μg/mL)	MIC_50_ (μg/mL)	MIC_90_ (μg/mL)
*S.* Typhi	*S.* Typhi_Tier_1 (n = 290)	3/83/14	0.015 to >4	0.25	4	99.6/-/0.4	1–64	4.70	4	8	100/-/-	0.03–1	0.20	0.12	0.5
	*S.* Typhi_Tier_2 (n = 191)	3/70/27	0.03 to >4	0.25	>4	100/-/0	1–16	5.75	4	8	100/-/-	0.06–1	0.10	0.12	0.5
	*S.* Typhi_Tier_3 (n = 1551)	3/55/42	0.007 to >4	0.5	4	100/-/0	0.5–16	5.65	4	8	100/-/-	0.015–1	0.22	0.25	0.5
	*S.* Typhi_cumulative (n = 2032)	3/60/37	0.007 to >4	0.5	>4	99.9/-/0.1	0.5–64	4.98	4	8	100/-/-	0.015–1	0.22	0.12	0.5
	Southern India (n = 834)	3/68/29	0.007 to >4	0.25	>4	100/-/0	0.5–16	5.31	4	8	100/-/-	0.015–1	0.24	0.25	0.5
	Northern India (n = 1089)	3/56/41	0.007 to >4	0.5	>4	100/-/0	0.5–16	5.56	4	8	100/-/-	0.03–1	0.21	0.12	0.5
	Western India (n = 71)	3/49/48	0.015 to >4	*	*	100/-/0	2–16	6.47	*	*	100/-/-	0.03–0.25	0.15	*	*
	Eastern India (n = 38)	5/42/53	0.03 to >4	*	*	97/-/3	2–64	7.42	*	*	100/-/-	0.06–0.5	0.16	*	*
*S.* Paratyphi A	SPA_Tier_1 (n = 21)	24/62/14	0.06 to >4	*	*	100/-/0	2–8	6.47	*	*	100/-/-	0.12–0.5	0.21	*	*
	SPA_Tier_2 (n = 44)	0/91/9	0.12 to >4	*	*	100/-/0	2–16	7.09	*	*	100/-/-	0.06–0.5	0.20	*	*
	SPA_Tier_3 (n = 276)	1/78/21	0.007 to >4	0.5	4	100/-/0	0.25–16	7.50	8	16	100/-/-	0.06–1	0.20	0.25	0.25
	SPA_cumulative (n = 341)	2/79/19	0.007 to >4	0.5	2	100/-/0	0.5–16	7.39	4	16	100/-/-	0.06–1	0.20	0.25	0.25
	Southern India (n = 115)	3/76/21	0.007 to >4	0.5	4	100/-/0	0.25–16	7.22	8	16	100/-/-	0.12–1	0.21	0.12	0.25
	Northern India (n = 208)	0/81/19	0.12 to >4	0.5	2	100/-/0	0.5–16	7.58	8	16	100/-/-	0.06–0.5	0.20	0.25	0.25
	Western India (n = 12)	33/67/0	0.06 to 0.5	*	*	100/-/0	2–8	5.5	*	*	100/-/-	0.12–0.5	0.17	*	*
	Eastern India (n = 6)	0/67/33	0.5 to 4	*	*	100/-/0	8	8	*	*	100/-/-	0.12–0.25	0.20	*	*

Abbreviations: MIC, minimum inhibitory concentration.

*MIC_50_/MIC_90_ was not calculated due to fewer isolates <100.

The MIC distribution across the 4 major geographical regions in India suggests that the *S.* Typhi MIC_50_ is 0.25 μg/mL for Southern India (Table 2) and is remarkably 1-fold dilution higher in the North. The MIC values for Western and Eastern India cannot be compared because the number of isolates was not significant. Overall, the MIC_90_ values for *S.* Typhi are comparatively higher than for *S.* Paratyphi A.

### Azithromycin Minimum Inhibitory Concentration

Among the 2373 clinical isolates of *S.* Typhi and *S.* Paratyphi A tested for azithromycin susceptibility using BMD, except for a single *S*. Typhi that was resistant, all *S*. Paratyphi A isolates were susceptible. As observed in Table 2, the azithromycin MIC ranged from 0.5 to 64 μg/mL for *S.* Typhi and 0.5 to 16 μg/mL for *S.* Paratyphi A. The azithromycin MIC distribution range with the highest proportion of *S.* Typhi and *S.* Paratyphi A isolates were 4–8 μg/mL and 8 μg/mL, respectively (Figure 2B). The cumulative MIC distribution showed 0.04% of the *S.* Typhi (*n* = 1) was resistant to azithromycin. In particular, up to 43% of the *S.* Typhi population had an MIC of ≤4 μg/mL, 34% had an MIC of 8 μg/mL, and 4.3% had an MIC of 16 μg/mL. In comparison to *S*. Typhi, *S.* Paratyphi A showed increased MIC of 8 μg/mL in 50% of isolates. The mean MIC of *S.* Typhi was 4.98 μg/mL, whereas *S.* Paratyphi A showed a higher mean MIC of 7.39 μg/mL, and up to 13% of isolates had an MIC of 16 μg/mL.

The Tier-wise distribution showed that *S.* Typhi isolates that belonged to Tier 2 had a higher mean MIC of 5.75 μg/mL, in comparison with Tier 1 (4.70 μg/mL) and Tier 3 (5.65 μg/mL). There was no variation in their MIC_50_ (4 μg/mL) and MIC_90_ (8 μg/mL) values across different settings. Compared with *S*. Typhi, *S*. Paratyphi A isolates from Tier 3 sites showed the highest mean MIC of 7.50 μg/mL, followed by Tier 2 (7.09 μg/mL) and Tier 1 (6.47 μg/mL). Region-specific MIC distribution of *S.* Typhi and *S.* Paratyphi A showed a higher mean MIC in Northern India, compared with Southern Indian region.

### Ceftriaxone Minimum Inhibitory Concentration

The MIC distribution of ceftriaxone showed that isolates of *S.* Paratyphi A were highly susceptible compared with *S.* Typhi. The MIC ranged from 0.015 to 1 μg/mL for *S.* Typhi, whereas it was 0.06–1 μg/mL for *S.* Paratyphi A. Cumulative data show that 44% of *S.* Typhi isolates had an MIC of 0.12 μg/mL. Most *S.* Paratyphi A isolates had an MIC distributed between 0.12 and 0.25 μg/mL, and no isolates were in the intermediate range (Figure 2C). Subset analysis of Tier-wise data did not show significant variations in the MIC variables for both *S.* Typhi and *S.* Paratyphi A. However, regional differences in the mean MIC were detected, for both *S.* Typhi and *S.* Paratyphi A, with higher mean MIC values in Southern India compared with other regions.

### Comparative Analysis of Disk Diffusion and Broth Microdilution Testing

**Figure 3.**
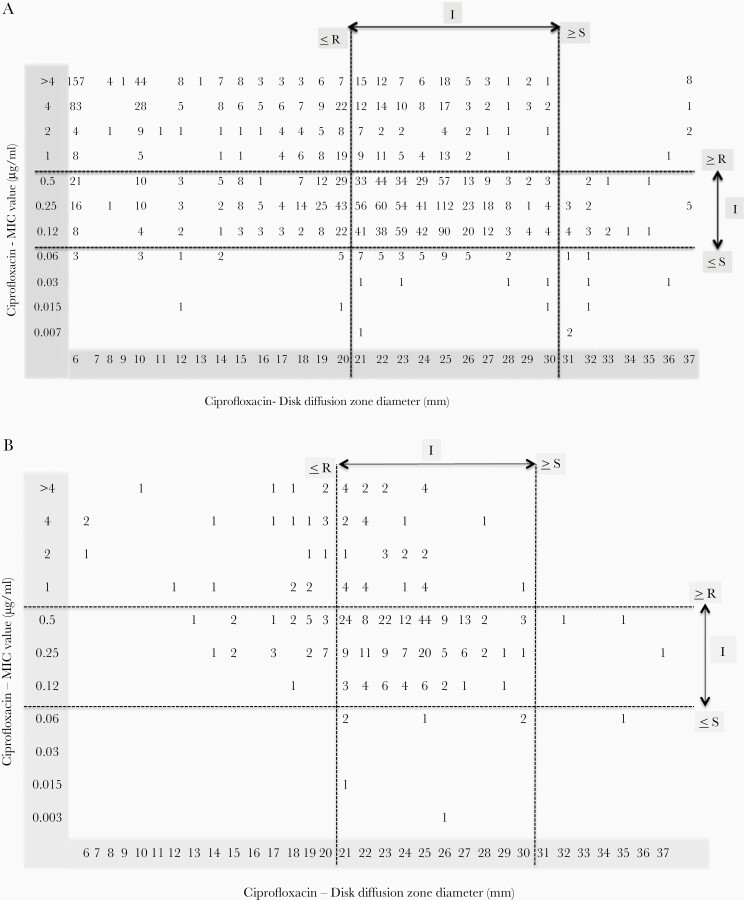

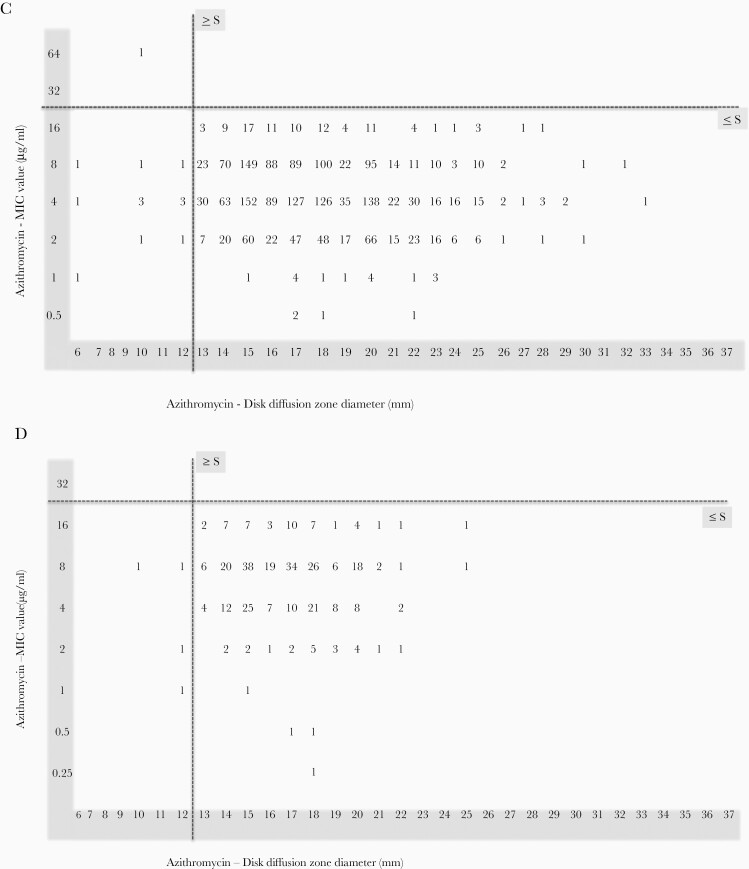

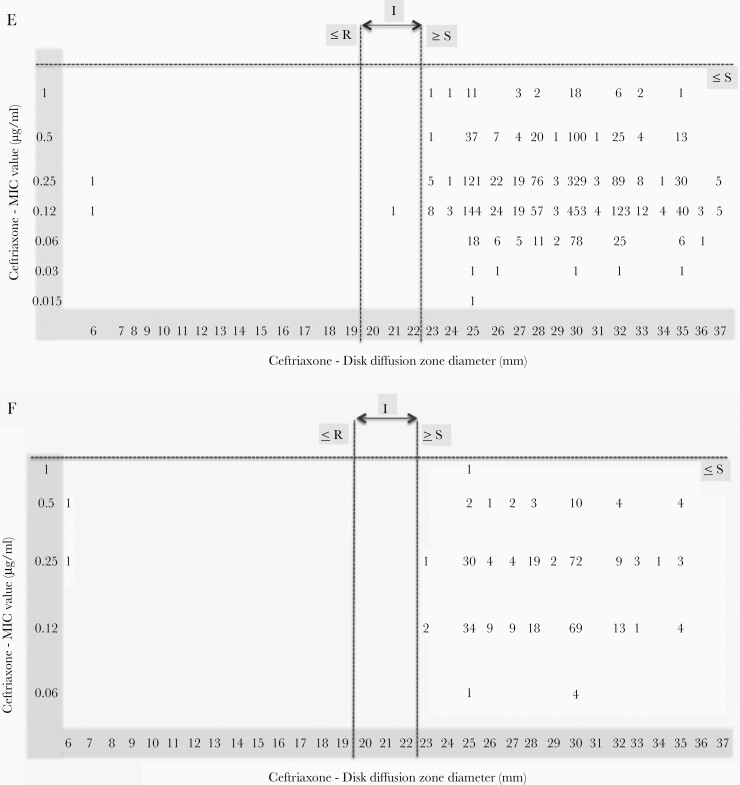
Relationship between zone of inhibition and minimum inhibitory concentration (MIC) in invasive isolates of *Salmonella* Typhi and *Salmonella* Paratyphi A included in this study. (A) Scatter plot of disk diffusion (DD) and MIC value of 2032 *S.* Typhi isolates tested against ciprofloxacin. (B) Scatter plot of DD and MIC value of 341 *S.* Paratyphi A isolates tested against ciprofloxacin. (C) Scatter plot of DD and MIC value of 2032 *S.* Typhi isolates tested against azithromycin. (D) Scatter plot of DD and MIC value of 341 *S.* Paratyphi A isolates tested against azithromycin. (E) Scatter plot of DD and MIC value of 2032 *S.* Typhi isolates tested against ceftriaxone. (F) Scatter plot of DD and MIC value of 341 *S.* Paratyphi A isolates tested against ceftriaxone.

Comparison between DD and MIC data were carried out for ciprofloxacin, azithromycin, and ceftriaxone (Figure 3A–F). For ciprofloxacin, several susceptible *S.* Typhi isolates by DD were found to be in the intermediate and resistant range when tested by MIC. Such discrepancies were not observed in the susceptibility profile of *S.* Paratyphi A. The overall concordance rates of DD and BMD for ciprofloxacin was 71.2% and 75.65% for *S.* Typhi and *S.* Paratyphi A, respectively. However, *S.* Typhi concordance rate of DD to BMD for intermediate was 82%, whereas it was less for susceptible (63.6%) and resistant (56.8%). Likewise, *S.* Paratyphi A concordance rate showed 57.6%, 82.7%, and 75% for susceptible, intermediate, and resistant, respectively. Also for ceftriaxone, it was 99.8% and 99.4% for *S.* Typhi and *S.* Paratyphi A, respectively.

For azithromycin, >95% concordance between DD and MIC was observed. In addition, among the 14 *S.* Typhi isolates found to be resistant by DD, only 1 isolate was confirmed as resistant by BMD. This discrepancy necessitates further confirmation of azithromycin by BMD testing. It is notable that 25% and 35% of the susceptible population of *S.* Typhi and *S.* Paratyphi A, respectively, by MIC (4–16 μg/mL) were found to have zone diameters between 13 and 15 mm (±3 mm of the susceptible cutoff). Differences of 2-fold dilution by MIC and zone diameter (DD being ±3 mm) within the susceptible criteria in a large number of tested isolates suggest that the azithromycin susceptibility profile may not be accurately determined by DD. Considering only MIC values, an MIC shift towards the susceptibility cutoff can be observed for both *S.* Typhi and *S.* Paratyphi A. It is notable that *S.* Paratyphi A showed a greater number of isolates towards higher MIC values when the criteria for azithromycin susceptibility testing for *S.* Typhi are extrapolated.

### Beta-Lactam/Beta-Lactamase Inhibitor

**Figure 4.**
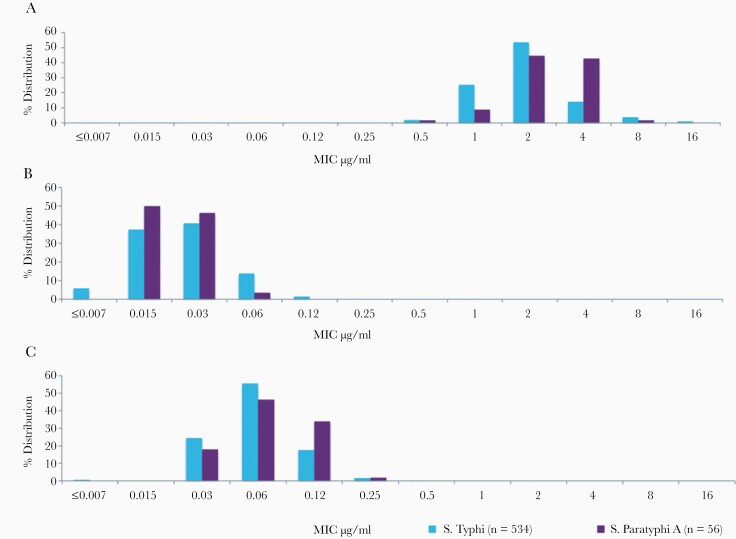
Histogram showing the distribution of minimum inhibitory concentration of (a) piperacillin/tazobactam, (b) cefepime/tazobactam, and (c) cefepime/zidebactam tested against *Salmonella* Typhi and *Salmonella* Paratyphi A.

All tested *S.* Typhi and *S.* Paratyphi A isolates were found to be susceptible to the classic first-generation BL/BLI-piperacillin/tazobactam. However, the distribution of MIC peaked at 1 μg/mL and 2 μg/mL for *S.* Typhi, whereas it was 2 μg/mL and 4 μg/mL for *S.* Paratyphi A. In contrast, the other 2 combinations of cefepime/tazobactam and cefepime/zidebactam showed greater *in vitro* activity against both *S.* Typhi and *S.* Paratyphi A. Cefepime/tazobactam and cefepime/zidebactam showed similar efficacy against *S.* Typhi, whereas for *S.* Paratyphi A, cefepime/tazobactam was significantly more efficacious compared with cefepime/zidebactam (Figure 4A–C). For *S.* Typhi, the mean MIC was lowest for cefepime/tazobactam and cefepime/zidebactam (0.06 μg/mL), whereas for piperacillin/tazobactam it was 2.38 μg/mL. Likewise, for *S.* Paratyphi A, the lowest mean MIC was observed for cefepime/tazobactam (0.04 μg/mL), followed by cefepime/zidebactam (0.07 μg/mL) and piperacillin/tazobactam (2.8 μg/mL).

## Discussion

The incidence rate and antimicrobial susceptibility pattern of laboratory-confirmed typhoid and paratyphoid cases were known only for a few endemic spots in India [4, 25]. Therefore, a pan-India study that encompasses a broader population and represents diverse geographical regions is necessary. The present study aided in the generation of such data by examining the susceptibility of 2032 *S.* Typhi and 341 *S.* Paratyphi A isolates, from across India, to ampicillin, chloramphenicol, co-trimoxazole, ciprofloxacin, ceftriaxone, and azithromycin. In addition, the *in vitro* efficacy of the classic BL/BLI piperacillin-tazobactam and newer BL/BLI cefepime-tazobactam and cefepime-zidebactam were determined for a subset of isolates.

We observed that >95% of *S.* Typhi and 100% *S.* Paratyphi A isolates were susceptible to the traditional first-line agents, ie, ampicillin, chloramphenicol, and cotrimoxazole. Likewise, a high susceptibility was noted for second-line agents, ceftriaxone and azithromycin, whereas <5% of isolates were susceptible to ciprofloxacin. Piperacillin-tazobactam and the other 2 newer agents (cefepime-tazobactam and cefepime-zidebactam) exhibited potent activity against all the tested isolates. The susceptibility pattern was consistent with all isolates collected across the geographical regions in India.

The present study confirms the declining trend in the prevalence of MDR *S.* Typhi in India, with a high susceptibility to first-line agents, as reported previously [4, 25, 26]. This observation is similar to the scenario prevailing in Nepal [26, 27], while Bangladesh and Pakistan continue to report MDR *S.* Typhi [28, 29]. The MDR reversal in India and Nepal could be attributed to the minimal prescription of first-line agents and, therefore, a lack of resistance selection pressure. In contrast, reduced ciprofloxacin susceptibility is on the rise in all 4 South Asian countries, pointing towards extensive use of this drug leading to the selection of nonsusceptible clones [26].

The present study results confirmed that ciprofloxacin should no longer be a drug of choice for treating typhoid fever. The value of ciprofloxacin in treating typhoid fever arises from multiple factors other than the *in vitro* activity. These include (1) convenience of oral therapy, (2) less frequent regimens (twice a day) due to its pharmacodynamic activity influenced by the area under concentration, (3) high intracellular concentration, (4) potent bactericidal activity, and (5) affordability [30]. Several reports described the ability of *Salmonella* species to become quinolone intermediate-susceptible/resistant, often through 1 or more mutations in type II topoisomerases (deoxyribonucleic acid [DNA] gyrase and DNA topoisomerase IV) and less commonly by acquiring plasmid-borne *qnr* genes [20, 30–32]. Mutations associated with DCS profile are S83F, S83Y, and D87N in *gyrA* and E84G, E84K, and S80I in *parC* [32]. Accumulation of stepwise mutation from single to double to triple mutations in *gyrA* and *parC* made the use of fluoroquinolones obsolete for typhoid management. Clinical implication of these mutations correlates well with estimated *in vitro* MIC levels, ie, isolates with single mutation displayed a low-level resistance with MIC of 0.25–0.38 μg/mL, whereas double/triple mutations displayed high-level resistance with MIC ranging from 12 to 32 μg/mL [20].

Both *S.* Typhi and *S.* Paratyphi A isolates tested in this study, showed high susceptibility to azithromycin and ceftriaxone, the current empirical therapy for typhoid management in India. However, a closer look at the data showed regional variations in ceftriaxone and azithromycin MIC profile. For instance, a higher mean azithromycin MIC was observed for north Indian isolates compared with south Indian isolates. On the contrary, typhoidal *Salmonella* isolates from South India showed a relatively higher mean ceftriaxone MIC compared with North India. Such variation could be due to differences in antibiotic prescription patterns between the regions, as previously reported [33].

Among the antibiotics that retained a >95% susceptibility rate in this study, azithromycin is known for attaining high intracellular concentration (50- to 100-fold higher than the serum level), although MICs are relatively on the higher side. However, for azithromycin, until 2015, there was no clinical breakpoint defined to interpret susceptibility for clinical isolates of typhoidal *Salmonella*. In earlier studies, an MIC of ≤16 μg/mL was used as an epidemiological cutoff for wild-type *Salmonella* spp [34]. In 2015, CLSI recommended azithromycin susceptibility breakpoints for *S.* Typhi (*S.* Paratyphi A was excluded). More importantly, unlike many other antimicrobial agents, CLSI has defined narrow susceptible and resistant breakpoints as ≥13 mm and ≤12 mm, respectively, for disc diffusion and ≤16 μg/mL and ≥32 μg/mL, respectively, for MIC. No intermediate category has been defined; therefore, the clinical strains that show zone diameters/MIC (±3-mm/1-tube dilution) closer to the susceptible breakpoint are problematic to interpret. Moreover, the appearance of a double zone is an additional challenge in accurately measuring the zone diameter [15]. The scatter plot generated in this study comparing zone diameters and MICs clearly highlights the substantial spread of zone diameters (for 30% of tested isolates) at the upper border of susceptible breakpoint (±3 mm). These gray zone of ±3-mm zone size may be recorded as false resistance or false susceptible because the testing criteria does not recommend a buffer zone with intermediate range between susceptible and resistant. These aforementioned factors contribute to interobserver variation resulting in the improper interpretation of the susceptibility report. Moreover, multiple clinical failures with azithromycin therapy have been reported in the recent past [35, 36]. The majority of the clinical failures documented to date are reported to be caused by isolates that were azithromycin susceptible *in vitro*. This raises the concern of low-level resistance, which might not be picked up by *in vitro* testing. Furthermore, a higher proportion of isolates with borderline susceptibility may be reflective of the abundant use of azithromycin in recent days for the treatment of typhoid fever [13]. Furthermore, there is a lack of correlation *in vitro* to *in vivo*, and because the azithromycin resides intracellularly at higher concentrations than at the systemic circulation, it is challenging to measure the precise activity. Considering these issues, it is highly critical to revisit the testing methodologies as well the clinical breakpoints.

In this scenario of fluoroquinolones being obsolete and several uncertainties associated with azithromycin, ceftriaxone appears to be the most viable treatment agent. However, the recent emergence of ceftriaxone-resistant, XDR *S.* Typhi in Pakistan raised a serious concern [17, 18]. It is important to note that the XDR *S.* Typhi outbreak strain belongs to the dominant H58 haplotype, which known for its propensity to spread geographically and therefore could potentially replace endemic clones when transferred. There is a great risk for transmission of such XDR *S.* Typhi clones to the neighboring countries as it has been documented earlier with H58 lineage [18], making it essential to explore alternative treatment options.

A clinical trial comparing mono versus combination therapy of azithromycin and ceftriaxone demonstrated a significantly shorter fever clearance time and bacterial elimination with the combination compared with a single regimen [12]. An additional study by Capoor et al [37] showed that carbapenems and tigecycline could potentially treat extended-spectrum beta-lactamase-producing MDR strains. However, clinical trial data were limited [37]. Another study documented poor clinical response to meropenem and highlighted the need for robust clinical data evaluating the efficacy of carbapenems for the treatment of typhoid fever [38]. In this scenario, intravenous, BL/BLI combinations could be used as alternative agents to treat Typhoid fever caused by XDR strains. The current study results showed potent *in vitro* activity of piperacillin-tazobactam, cefepime-tazobactam, and cefepime-zidebactam as well, warranting further studies including clinical evaluation. Among these, the antibacterial action of cefepime-zidebactam is driven by β-lactam enhancer action.

## Conclusions

In summary, we show that Indian typhoidal *Salmonella* isolates are generally nonsusceptible to ciprofloxacin, whereas they have regained susceptibility to first-line agents. Although the tested isolates were highly susceptible to azithromycin, reports of azithromycin treatment failure even in the case of infections caused by susceptible isolates question the testing interpretation. There was no appreciable resistance to ceftriaxone in this study. The potent activity against tested isolates, observed with BL-BLIs supports further evaluation of these agents for the treatment of XDR and complicated Typhoid cases. Continuous AMR surveillance and constant re-evaluation of empiric antimicrobial therapy needs to be implemented to facilitate evidence-based national policy decisions and practice.

## Figures and Tables

**Table:** 

• Manikandan	Srinivasan	Christian Medical College, Vellore
• Anita Shirley	David	Christian Medical College, Vellore
• Atrayee	Nag	Christian Medical College, Vellore
• Kulandaipalayam Natarajan	Sindhu	Christian Medical College, Vellore
• Malathi	Murugesan	Christian Medical College, Vellore
• Nirmal	Kumar	Christian Medical College, Vellore
• Prabakhar D	Moses	Christian Medical College, Vellore
• Ranjith Kumar	R.	Christian Medical College, Vellore
• Sathyapriya	Subramaniam	Christian Medical College, Vellore
• Swathi	Vajja	Christian Medical College, Vellore
• Ashish	Bavdekar	KEM Hospital & Research Centre, Pune
• Ankita	Shrivastava	KEM Hospital & Research Centre, Pune
• Bharat	Choudhari	KEM Hospital & Research Centre, Pune
• Sandeep	Bhujbal	KEM Hospital & Research Centre, Pune
• Sanjay	Juvekar	KEM Hospital & Research Centre, Pune
• Sonali	Sanghavi	KEM Hospital & Research Centre, Pune
•Tathagata	Bhattacharjee	KEM Hospital & Research Centre, Pune
• Shanta	Dutta	ICMR- National Institute of Cholera & Enteric Diseases, Kolkata
• Debottam	Pal	ICMR- National Institute of Cholera & Enteric Diseases, Kolkata
• Jayanta	Saha	ICMR- National Institute of Cholera & Enteric Diseases, Kolkata
• Meghna	Bannerjee	ICMR- National Institute of Cholera & Enteric Diseases, Kolkata
• Nandini	Chakrabarty	ICMR- National Institute of Cholera & Enteric Diseases, Kolkata
• Pranab	Chatterjee	ICMR- National Institute of Cholera & Enteric Diseases, Kolkata
• Suman	Kanungo	ICMR- National Institute of Cholera & Enteric Diseases, Kolkata
• Tania	Pan	ICMR- National Institute of Cholera & Enteric Diseases, Kolkata
• Temsunaro	Rongsen-Chandola	Centre for Health Research & Development-Society for Applied Studies, New Delhi
• Alok	Arya	Centre for Health Research & Development-Society for Applied Studies, New Delhi
•Ananya	Tupaki- Sreepurna	Centre for Health Research & Development-Society for Applied Studies, New Delhi
• Ankita	Dutta	Centre for Health Research & Development-Society for Applied Studies, New Delhi
• Aparna	Chakravarty	Hamdard Institue of Medical Sciences and Research, New Delhi
• Bireshwar	Sinha	Centre for Health Research & Development-Society for Applied Studies, New Delhi
• Chandra Mohan	Kumar	Hamdard Institue of Medical Sciences and Research, New Delhi
• Deepak	More	Centre for Health Research & Development-Society for Applied Studies, New Delhi
• Mohammed	Aslam	Centre for Health Research & Development-Society for Applied Studies, New Delhi
• Nidhi	Goyal	Centre for Health Research & Development-Society for Applied Studies, New Delhi
• Ashita	Singh	Chinchpada Christian Hospital, Nandurbar
• Alice	Hepzibah	Chinchpada Christian Hospital, Nandurbar
• Miriam Thankam	George	Chinchpada Christian Hospital, Nandurbar
• Sheena	Evelyn Ebenezer	Duncan Hospital, Raxaul
• Mathew	Santosh Thomas	Duncan Hospital, Raxaul
• Anna	P. Alexander	Lady Willingdon Hospital, Manali
• Christina	Dhas Sankhro	Lady Willingdon Hospital, Manali
• Dennis	Martin David	Lady Willingdon Hospital, Manali
• Pradeep	Zachariah	Lady Willingdon Hospital, Manali
• Roshine Mary	Koshy	Makunda Christian Leprosy & General Hospital, Karimganj
• Jasmine Susan	Koshy	Makunda Christian Leprosy & General Hospital, Karimganj
•Kenningpeule	D. Haikube	Makunda Christian Leprosy & General Hospital, Karimganj
• Shajin	T.	Makunda Christian Leprosy & General Hospital, Karimganj
• Madhu	Gupta	Post Graduate Institute of Medical Education & Research, Chandigarh
• Adarsh	Bansal	Post Graduate Institute of Medical Education & Research, Chandigarh
• Akashdeep	Singh Chauhan	Post Graduate Institute of Medical Education & Research, Chandigarh
• Atul	Sharma	Post Graduate Institute of Medical Education & Research, Chandigarh
• Bal		Post Graduate Institute of Medical Education & Research, Chandigarh
• Gopal	Bhardwaj	Post Graduate Institute of Medical Education & Research, Chandigarh
• Gurinderjeet	Singh	Post Graduate Institute of Medical Education & Research, Chandigarh
• Krishna	Chaudhary	Post Graduate Institute of Medical Education & Research, Chandigarh
• Nayantara		Post Graduate Institute of Medical Education & Research, Chandigarh
• Nitasha		Post Graduate Institute of Medical Education & Research, Chandigarh
• Rajesh	Kumar	Post Graduate Institute of Medical Education & Research, Chandigarh
• Sadbhavna	Pandit	Post Graduate Institute of Medical Education & Research, Chandigarh
• Saroj Kumar	Rana	Post Graduate Institute of Medical Education & Research, Chandigarh
• Shankar	Prinja	Post Graduate Institute of Medical Education & Research, Chandigarh
• Vidushi	Mahajan	Post Graduate Institute of Medical Education & Research, Chandigarh
• Vishal	Guglani	Post Graduate Institute of Medical Education & Research, Chandigarh
• Vivek	Sagar	Post Graduate Institute of Medical Education & Research, Chandigarh
• Dasaratha Ramaiah	Jinka	Rural Development Trust Hospital, Bathalapalli, Andhra Pradesh
• Raghuprakash	Reddy Nayakanti	Rural Development Trust Hospital, Bathalapalli, Andhra Pradesh
• Arti	Kapil	All India Institute of Medical Sciences, New Delhi
• Bimal Kumar	Das	All India Institute of Medical Sciences, New Delhi
• Manish	Soneja	All India Institute of Medical Sciences, New Delhi
• Rakesh	Lodha	All India Institute of Medical Sciences, New Delhi
• Seema	Sood	All India Institute of Medical Sciences, New Delhi
• Karnika	Saigal	Chacha Nehru Bal Chikitsalaya, Delhi
• Arnab	Ghosh	Chacha Nehru Bal Chikitsalaya, Delhi
• Deepika	Gupta	Chacha Nehru Bal Chikitsalaya, Delhi
• Diganta	Saikia	Chacha Nehru Bal Chikitsalaya, Delhi
• Mamta	Senger	Chacha Nehru Bal Chikitsalaya, Delhi
• Maria	Thomas	Christian Medical College, Ludhiana
• Aroma	Oberoi	Christian Medical College, Ludhiana
• Dhruva	Ghosh	Christian Medical College, Ludhiana
• Shalini	Anandan	Christian Medical College, Vellore
• Anushree	Amladi	Christian Medical College, Vellore
• Baby	S. Abirami	Christian Medical College, Vellore
• Dhiviya	P. M. Sethuvel	Christian Medical College, Vellore
• Yamuna	D. Bhakthavatchalam	Christian Medical College, Vellore
• Sulochana Putli Bai	Perumal	Kanchi Kamakoti CHILDS Trust Hospital, Chennai
• S. Balasubramanian		Kanchi Kamakoti CHILDS Trust Hospital, Chennai
• S. Senthilnathan		Kanchi Kamakoti CHILDS Trust Hospital, Chennai
• Pallab	Ray	Post Graduate Institute of Medical Education & Research, Chandigarh
• Archana	Angrup	Post Graduate Institute of Medical Education & Research, Chandigarh
• Manisha	Biswal	Post Graduate Institute of Medical Education & Research, Chandigarh
• Savitha	Nagaraj	St Johns Medical College, Bengaluru
• Anirudh	V.	St Johns Medical College, Bengaluru
• Lissy	P. J.	St Johns Medical College, Bengaluru
• Poornima	R. N.	St Johns Medical College, Bengaluru
• Prasanna	Kumar A. R.	St Johns Medical College, Bengaluru
• Ratnamala	Choudhury	St Johns Medical College, Bengaluru
• Sanju	Jose	St Johns Medical College, Bengaluru
• Vani	Channareddy	St Johns Medical College, Bengaluru
• Jayanthi	Shastri	Topiwala National Medical College & BYL Nair Charitable Hospital, Mumbai
• Anuradha	De	Topiwala National Medical College & BYL Nair Charitable Hospital, Mumbai
• Mala	Kaneria	Topiwala National Medical College & BYL Nair Charitable Hospital, Mumbai
• Sachee	Agrawal	Topiwala National Medical College & BYL Nair Charitable Hospital, Mumbai
• Sushma	Mallik	Topiwala National Medical College & BYL Nair Charitable Hospital, Mumbai
• Gagandeep	Kang	Christian Medical College, Vellore
• Agila K.	Pragasam	Christian Medical College, Vellore
• Arun	S. Karthikeyan	Christian Medical College, Vellore
• Balaji	Veeraraghavan	Christian Medical College, Vellore
• Dilesh Kumar	A.	Christian Medical College, Vellore
• Jacob	John	Christian Medical College, Vellore
• Jobin	J. John	Christian Medical College, Vellore
• Karthikeyan	Ramanujam	Christian Medical College, Vellore
• Nayana	P. Nair	Christian Medical College, Vellore
• Nikhil	Sahai	Christian Medical College, Vellore
• Prasanna	Samuel	Christian Medical College, Vellore
• Reshma	Raju	Christian Medical College, Vellore
• Santhosh Kumar	G.	Christian Medical College, Vellore
• Sidhartha	Giri	Christian Medical College, Vellore
• Swathi Krishna	N.	Christian Medical College, Vellore
• Venkata Raghava	Mohan	Christian Medical College, Vellore

## Notes

***Acknowledgments.*** We thank Duncan Steele, Megan Carey, Supriya Kumar, and the Bill & Melinda Gates Foundation for their technical support and review of the manuscript. We also thank Nicholas Grassly (Imperial College London) for assistance with study design and research proposal development.

***Financial support.*** This work was funded by grants from Bill & Melinda Gates Foundation, USA (Investment ID INV-009497 OPP1159351) for the Project “National Surveillance System for Enteric Fever in India.”

***Supplement sponsorship.*** This supplement is sponsored by the Christian Medical College Vellore Association.

***Potential conflicts of interest.*** All authors: No reported conflicts. All authors have submitted the ICMJE Form for Disclosure of Potential Conflict of Interest.
